# Supplementary material for: Regulation of Adipose Progenitor Cell Expansion in a Novel Micro-Physiological Model of Human Adipose Tissue Mimicking Fibrotic and Pro-Inflammatory Microenvironments
Source: Cells. 2022 Sep 7;11(18):2798. doi: 10.3390/cells11182798 (PMC9496930; doi:10.3390/cells11182798)
Supplement: Supplementary file 1 [file cells-11-02798-s001.zip › cells-1857469-supplementary.pdf]

Supplementary information

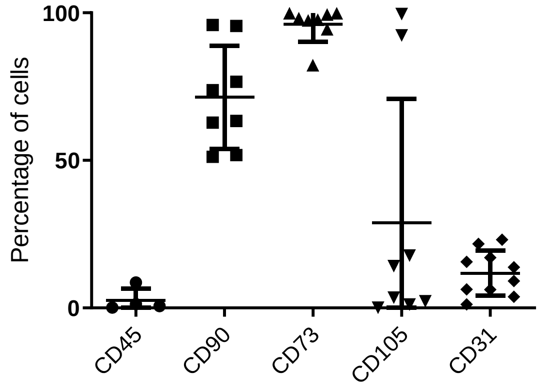

Supplementary Figure S1*:* Immunophenotype of adherent cells

ExAdEx models expanded for 2 weeks *in vitro* were digested with collagenase digestion and adherent cells were identified as APCs thanks to their immunophenotype. Cells were fixed with 4% paraformaldehyde for 15 min then stained for FACS analysis with CD105 (Clone BA5b/FITC; Ozyme, France), CD90 (Clone 1H4/APC; Molecular probe), CD73 (Clone HTF-1/PE; Invitrogen, France), CD31 (Clone WM59/PE; BD Biosciences) or CD45 (Clone HI30/PE; BD Biosciences). The percentage of cells is given. It is interesting to note a percentage of CD31^{+}cells, reflecting the maintenance of endothelial cells, and of CD45+ cells, suggesting the maintenance of some hematopoietic cells, in the ExAdEx model. Detection of CD68 positive cells by confocal microscopy supports this hypothesis (not shown).

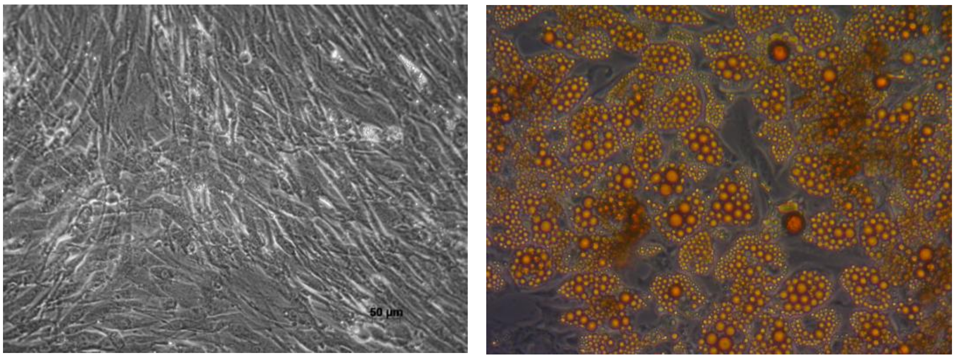

Supplementary Figure S2*:* Adipogenic capacity of adherent cells. Adherent cells were maintained in EGM (Cat: CC-4133; PromoCell, Germany) until they reach cell confluence, and then the medium was supplemented with1 µM Rosiglitazone; 2 µM T3; 2,5 µg/ml insulin and 250 µM Dexaméthasone ; 500

μM IBMX for the first 3 days only for 2 weeks. Then, cells were fixed and stained with Oil Red O for lipid droplets.

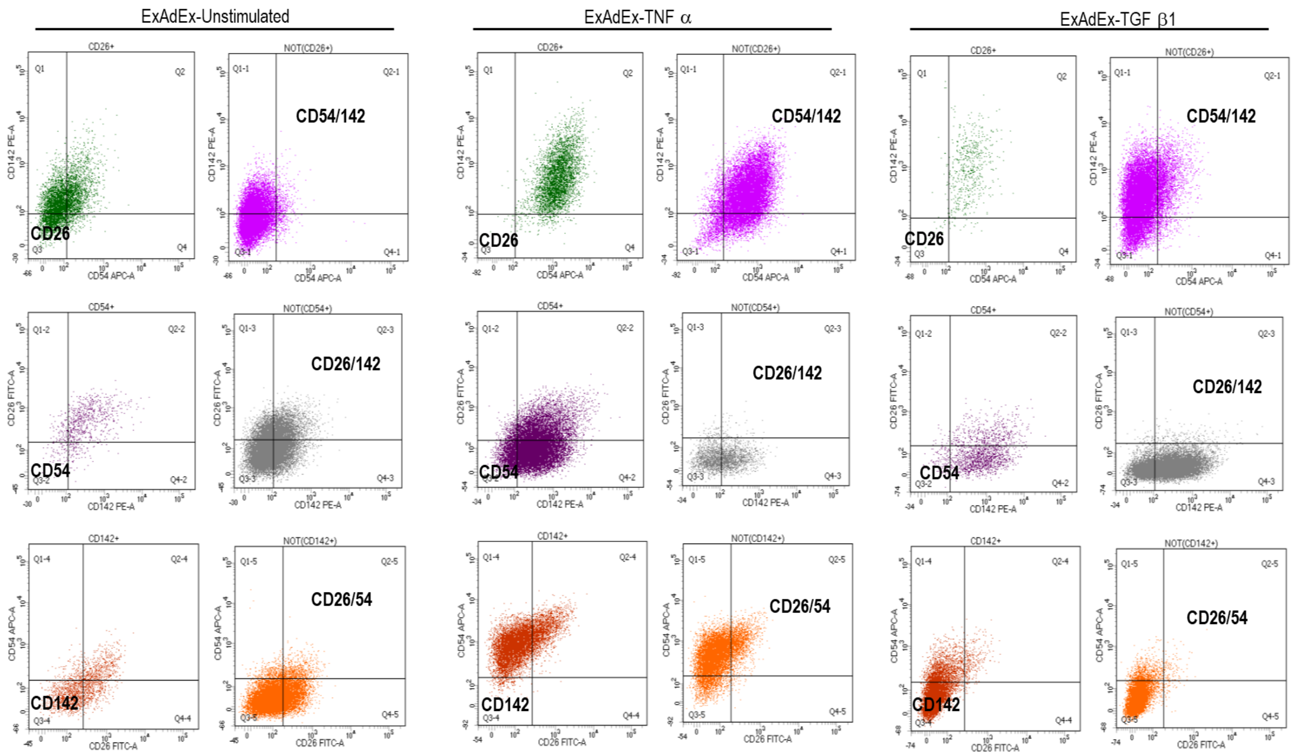

Supplementary Figure S3: Gating used to analyse percentages of APC subpopulations in unstimulated, TNFα– and TGFβ1-treated ExAdEx models.

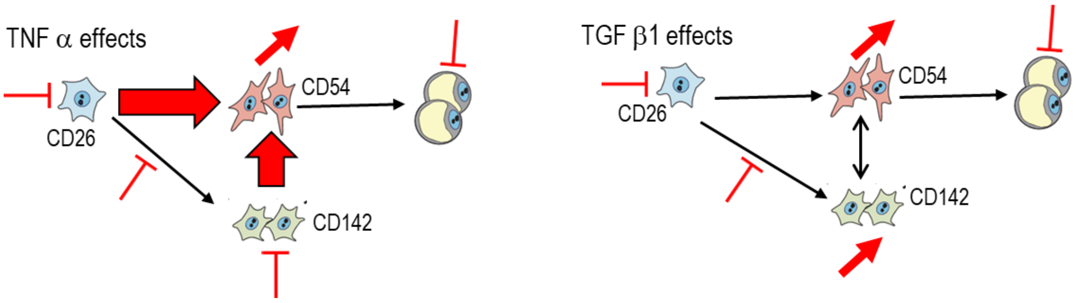

Supplementary Figure S4: A schema that summarizes the impacts of inflammatory and fibrotic micro-environments on APC populations**.**

Table S1

Target mRNA	Forward 5’-3’	Reverse 5’-3’
TBP	ACGCCAGCTTCGGAGAGTTC	CAAACCGCTTGGGATTATATTCG
CD26/DPP4	SinoBiological HP100649	SinoBiological HP100649
CD54/ICAM1	SinoBiological HP100384	SinoBiological HP100384
MMP14	CTCTCTTCTGGATGCCCAAT	ACCTCCGTCTCCTCCTCAGT
INHBA	GGGAGAACGGGTATGTGGAGAT	GCTGTTCCTGACTCGGCAAA
PLIN1	ACCATCTCCACCCGCCTC	GATGGGAACGCTGATGCTGT
GLUT4	CAT TCC TTG GTT CAT CGT G	ATA GCC TCC GCA ACA TAC

Note: Sequences of CD26/DPP4 and CD54/ICAM1 primers purchased at SinoBiological are not available
